# Transcriptional regulatory networks controlling taste and aroma quality of apricot (*Prunus armeniaca* L.) fruit during ripening

**DOI:** 10.1186/s12864-019-5424-8

**Published:** 2019-01-15

**Authors:** Qiuyun Zhang, Chao Feng, Wenhui Li, Zehui Qu, Ming Zeng, Wanpeng Xi

**Affiliations:** 1 College of Horticulture and Landscape Architecture, Southwest University, Chongqing, 400716 People’s Republic of China; 2 Key Laboratory of Plant Resources Conservation and Sustainable Utilization, South China Botanical Garden, Chinese Academy of Sciences, Guangzhou, 510650 People’s Republic of China; 3 Agriculture National Fruit Tree Germplasm Repository, Xinjiang Academy of Agricultural Sciences, Luntai, Xinjiang, 841600 People’s Republic of China; 4 College of Computer and Information Sciences, Southwest University, Chongqing, 400716 People’s Republic of China

**Keywords:** Apricot (*Prunus armeniaca* L.), RNA-Seq, Sugars, Organic acids, Aroma volatiles, Transcriptome, Gene network

## Abstract

**Background:**

Taste and aroma, which are important organoleptic qualities of apricot (*Prunus armeniaca* L.) fruit, undergo rapid and substantial changes during ripening. However, the associated molecular mechanisms remain unclear. The goal of this study was to identify candidate genes for flavor compound metabolism and to construct a regulatory transcriptional network.

**Results:**

We characterized the transcriptome of the ‘Jianali’ apricot cultivar, which exhibits substantial changes in flavor during ripening, at 50 (turning), 73 (commercial maturation) and 91 (full ripe) days post anthesis (DPA) using RNA sequencing (RNA-Seq). A weighted gene co-expression network analysis (WGCNA) revealed that four of 19 modules correlated highly with flavor compound metabolism (*P* < 0.001). From them, we identified 1237 differentially expressed genes, with 16 intramodular hubs. A proposed pathway model for flavor compound biosynthesis is presented based on these genes. Two *SUS1* genes, as well as *SPS2* and *INV1* were correlated with sugar biosynthesis, while *NADP-ME4*, two *PK-like* and mitochondrial energy metabolism exerted a noticeable effect on organic acid metabolism. *CCD1* and *FAD2* were identified as being involved in apocarotenoid aroma volatiles and lactone biosynthesis, respectively. Five sugar transporters (*Sweet10*, *STP13*, *EDR6*, *STP5.1, STP5.2*), one aluminum-activated malate transporter (*ALMT9*) and one ABCG transporter (*ABCG11*) were associated with the transport of sugars, organic acids and volatiles, respectively. Sixteen transcription factors were also highlighted that may also play regulatory roles in flavor quality development.

**Conclusions:**

Apricot RNA-Seq data were obtained and used to generate an annotated set of predicted expressed genes, providing a platform for functional genomic research. Using network analysis and pathway mapping, putative molecular mechanisms for changes in apricot fruit taste and aroma during ripening were elucidated.

**Electronic supplementary material:**

The online version of this article (10.1186/s12864-019-5424-8) contains supplementary material, which is available to authorized users.

## Background

Apricot (*Prunus armeniaca*) is one of the most economically important Rosaceae stone fruit crops, and provides a valuable source of nutrients and phytochemicals for the human diet [1]. In China, apricots are mainly cultivated in northern areas, especially in Xinjiang, which account for 62% of the total production of the country. As one of the primary centers of apricot domestication, nearly 200 varieties are grown in this region and they collectively show a wide diversity of fruit qualities [2]. Among these, the ‘Jianali’ cultivar is one of the most popular cultivars, due to consumer preference for its flavor, color and overall quality, it is a good material for studying the related quality traits. As one of the important organoleptic quality, characteristic flavor of apricot fruit derives from the combination of taste components and aromatic volatiles, it is mainly provided by sucrose, malic acid and characteristic aroma volatiles, such as β-ionone and γ-decalactone [3, 4].

RNA sequencing (RNA-Seq), based in next generation sequencing (NGS), is an attractive approach for gene discovery and functional analysis, it facilitates transcriptome studies in non-model organisms [5]. This technology has been used to examine gene function in many fruit crops; however, to date there are only a few published transcriptome studies of apricot fruit. These have focused on pericarp tissue development [6], mining of simple sequence repeat (SSR) markers [7], the identification of genes associated with pistil abortion [8], comparative analysis of fruit development in interspecific and intraspecific hybrids [9], developing embryos [10], oil accumulation in seed kernels [11], and endocarp cleaving [12]. At present, the apricot genome sequence is not publicly available and related genomic resources, including information to enhance molecular breeding of apricot fruit, are somewhat limited.

Fruit ripening is a genetically programmed process that is physiologically and biochemically irreversible [13]. It involves numerous metabolic and molecular changes that influence fruit qualities such as color, texture and flavor, many of which have been shown to be related to alterations in the activity of specific enzymes or complete pathways [14–16]. These lead to the accumulation of specific soluble sugars, organic acids, aroma volatiles, and hence fruit flavor is tightly linked to the ripening regulatory networks [17, 18].

To date, although progress has been made in understanding the changes in flavor compounds in apricot, less is known about the expression levels of flavor-related genes during apricot fruit ripening [19]. Several studies have also covered the discovery of ripening-related genes of apricots, but few genes were identified, and especially the metabolic regulation underlying fruit ripening is poorly understood [6, 17, 20]. In this study, we monitored changes in flavor compounds of ‘Jianali’ fruit during ripening and profiled the transcriptome using RNA-Seq, integrated transcriptome and metabolome-derived data to propose a biosynthetic pathway for compounds that contribute to flavor, identify the specific genes and putative transcription factors involved in these processes through network analysis, and ultimately construct a putative regulatory network controlling flavor quality development in apricot fruit.

## Results

### Transcriptome profiles during fruit ripening

**Fig. 1:**
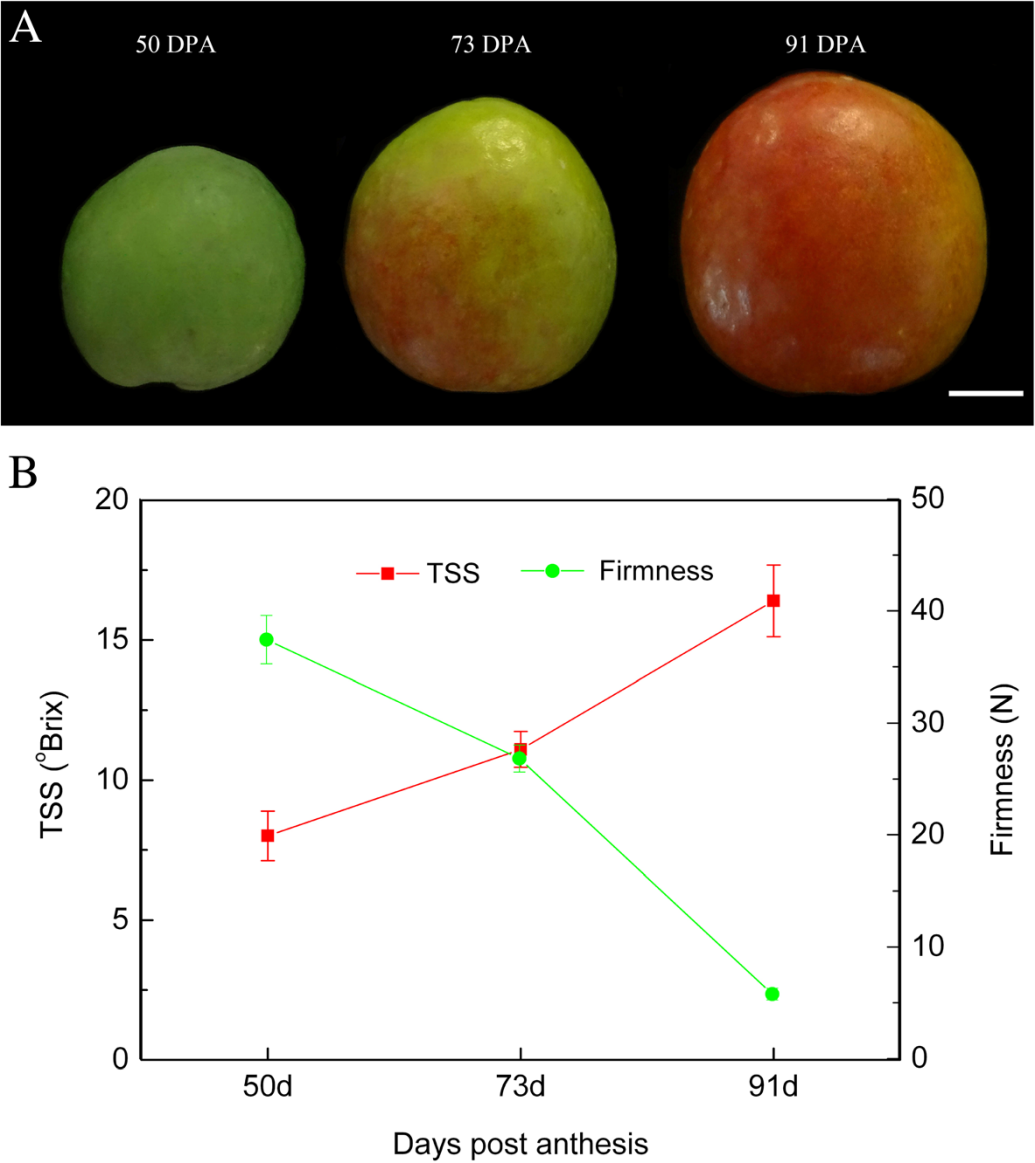
Apricot fruit samples used for RNA-sequencing and their basic quality changes through ripening. **a** 50, 73 and 91 days post anthesis (DPA), white bar = 1 cm. **b** Changes in total soluble solids (TSS) and firmness

Apricot fruit peels from the ‘Jianali’ cultivar at three ripening stages (turning, S1, 50 DPA; commercial maturation, S2, 73 DPA; full ripe, S3, 91 DPA) were subjected to RNA-Seq analysis (Fig. 1a). During the ripening process, total soluble solids (TSS) increased from 8 ^o^Brix to 16.4 ^o^Brix and firmness decreased from 37.43 N to 5.78 N (Fig. 1b), suggesting that the three stages represent the complete ripening process of ‘Jianali’ fruit and the fruit ripened normally during the period.

During the preprocessing of the RNA-Seq data, adaptor reads, ambiguous reads and low-quality reads were removed, resulting in an average of 2.36 gigabases (Gb) clean reads (Q_20_ > 97%) per library (Additional file 1). The reproducibility of the data was indicated by principal component analysis (PCA) model (Additional file 2), further supporting the validity of the experimental design and RNA-Seq data.

**Fig. 2:**
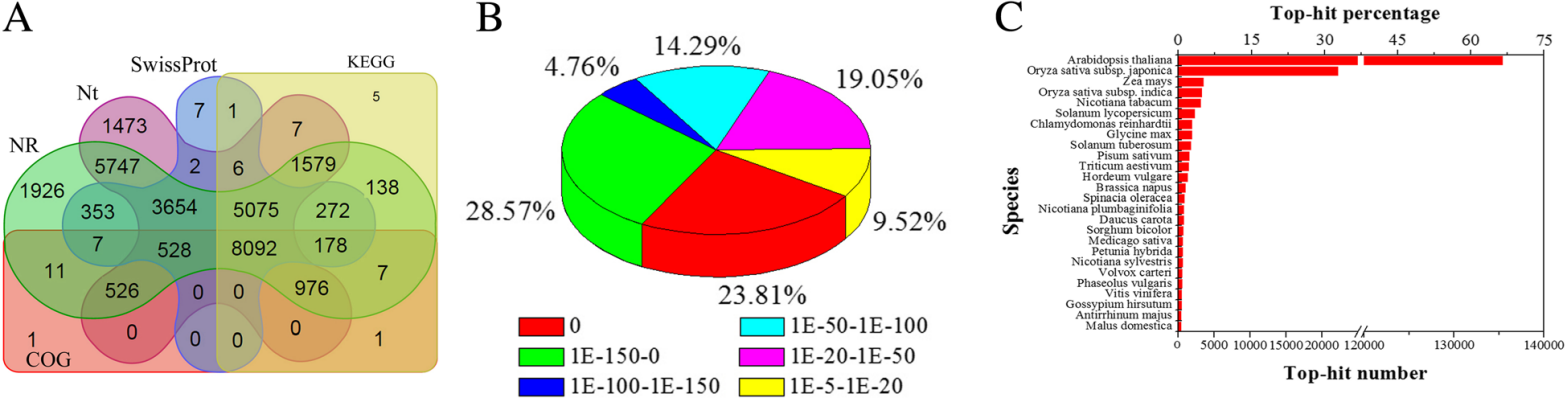
Summary of homology searches for apricot unigenes. **a** Venn diagram showing the number of unigenes annotated by BLASTx with an *E*-value threshold of 10^− 5^ against protein databases. The numbers in the circles indicate the number of unigenes annotated by single or multiple sequence databases, **b** *E*-value distribution of the top BLASTx hits against the NR database for each unigene, **c** Number and percentage of unigenes matching the 26 top species using a BLASTx search of the NR database

After assembly, 37,430 unigenes were generated from the nine libraries (Additional file 3), of which 30,566 genes, (~ 82%) were annotated as they showed a significant hit in the NCBI non-redundant database (NR), the Nucleotide Sequence Database (Nt), the Swiss-Prot proteins database, the Kyoto Encyclopedia of Genes and Genomes database (KEGG) and the Clusters of Orthologous Groups of proteins database (COG) databases. A total of 8092 sequences had the same homolog in all the databases (Fig. 2a). Based on the *E*-value distribution, 90% (29,065 unigenes) of the mapped and translated sequences showed homology to proteins in the NR database (*E*-value < 10^− 20^) (Fig. 2b). Approximately 80% of the unigenes could be annotated with sequences from the 5 top-hit species (Fig. 2c), and GO terms (for molecular function, cellular component and biological process) were assigned to 18,572 unigenes. Of the sub-categories, gene associated with the ‘metabolic process’ and ‘catalytic activity’ showed significant changes in expression during fruit ripening (Additional file 4).

### Differential expression genes (DEGs) and weighted gene co-expression network analysis (WGCNA)

**Fig. 3:**
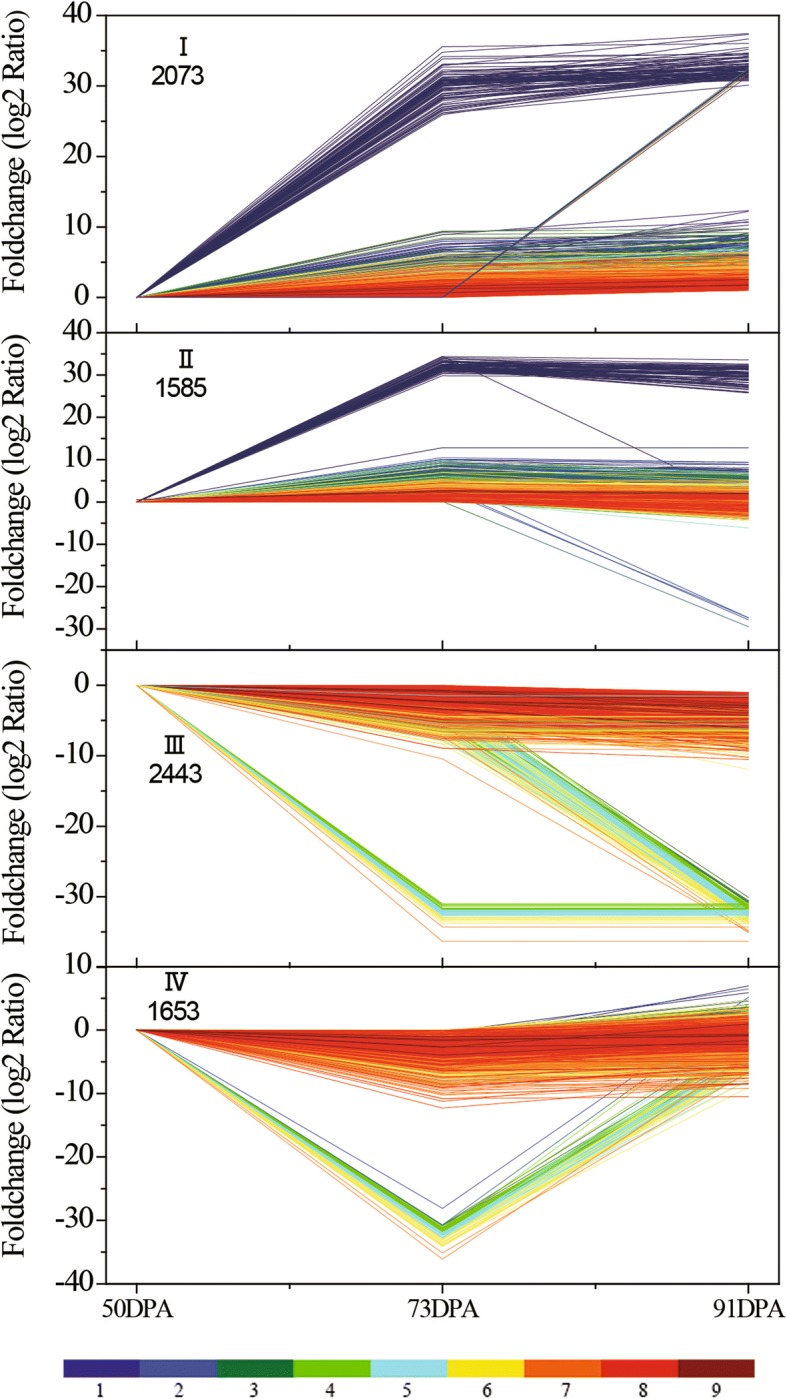
Unigene expression profiles during apricot fruit ripening. Four expression profiles are shown, with I and III indicating unigenes with up-regulated and down-regulated expression, respectively, and II and IV indicating those with up- and then down-regulated, and down- and then up-regulated expression, respectively. The 9 different colored lines show the absolute expression level at 50 DPA, with the FPKM values 0–0.1, 0.1–0.7, 0.7–2, 2–4, 4–8, 8–20, 20–100, 100–1000, 1000–12,500 represented by colors 1 to 9, respectively

During ripening, 37,430 unigenes were expressed, with 4555, 6173 and 1880 showing differential expression between S1 and S2, S1 and S3, and S2 and S3, respectively. Of these, we categorized 7754 differentially expressed unigenes into four groups (I-IV) according to their expression profiles, and these contained 2703, 1585, 2443 and 1653 unigenes, respectively. Groups I and III were defined as containing up-regulated and down-regulated genes, respectively, while genes in groups II and IV showed up- then down-regulation, and vice versa, respectively (Fig. 3).

**Fig. 4:**
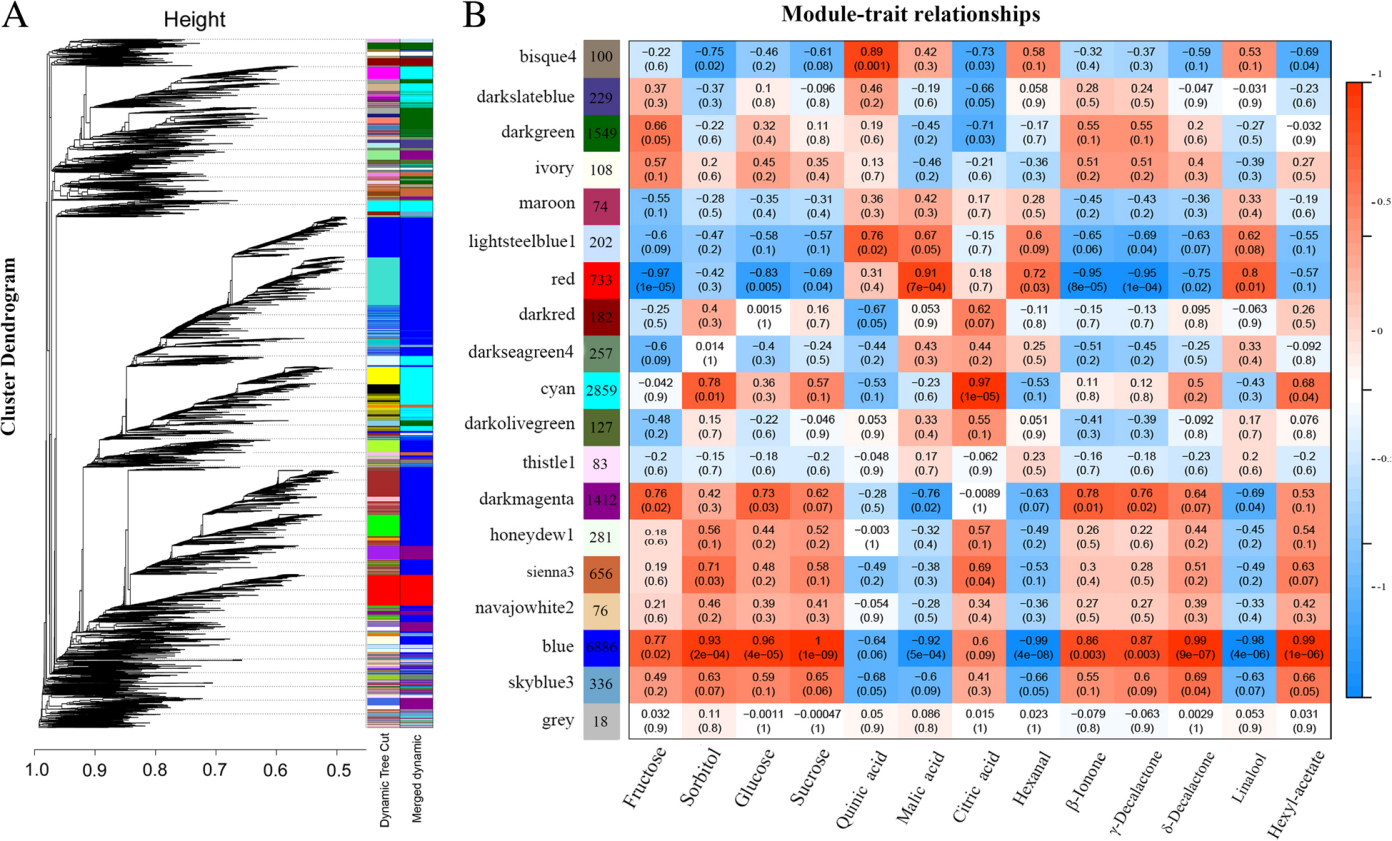
Co-expression network analysis of apricot transcriptomes during fruit ripening. **a** Hierarchical cluster tree showing 19 modules of co-expressed genes. Each leaf in the tree represents one gene. **b** Module-flavor correlations and corresponding *p*-values. The left panel shows 19 modules and the number of genes in each module. The right panel is a color scale for module trait correlation from − 1 to 1

A WGCNA was performed using the 16,168 unigenes that had a Fragments Per Kilobase of transcript per Million mapped reads (FPKM) value > 1. Genes were comprised of nineteen co-expression modules (Fig. 4a), of which four (blue, cyan, red, bisque) showed a significant association with flavor compounds changes during ripening (Fig. 4b). The content of fructose was highly negatively correlated with gene expression in the ‘red’ module, with a coefficient of − 0.97 (*P* = 1 × 10^− 5^). Sorbitol, glucose and sucrose showed a positive correlation with transcript abundance of genes in the ‘blue’ module, and malic acid content was highly positively correlated with the ‘red’ module, but negatively correlated with the ‘blue’ module. β-Ionone (*r* = 0.95, *P* = 8 × 10^− 5^) and γ-decalactone showed a significant positive correlation with the ‘blue’ module, but a negative correlation with the ‘red’ module. Finally, δ-dacalactone was highly positively correlated with the ‘blue’ module. Based on correlation (*r* > 0.91) between genes among the four modules and these flavor compounds, we identified that a total of 1237 structural genes and 16 transcription factors were related to flavor compound metabolism.

### Genes related to soluble sugar metabolism

**Fig. 5:**
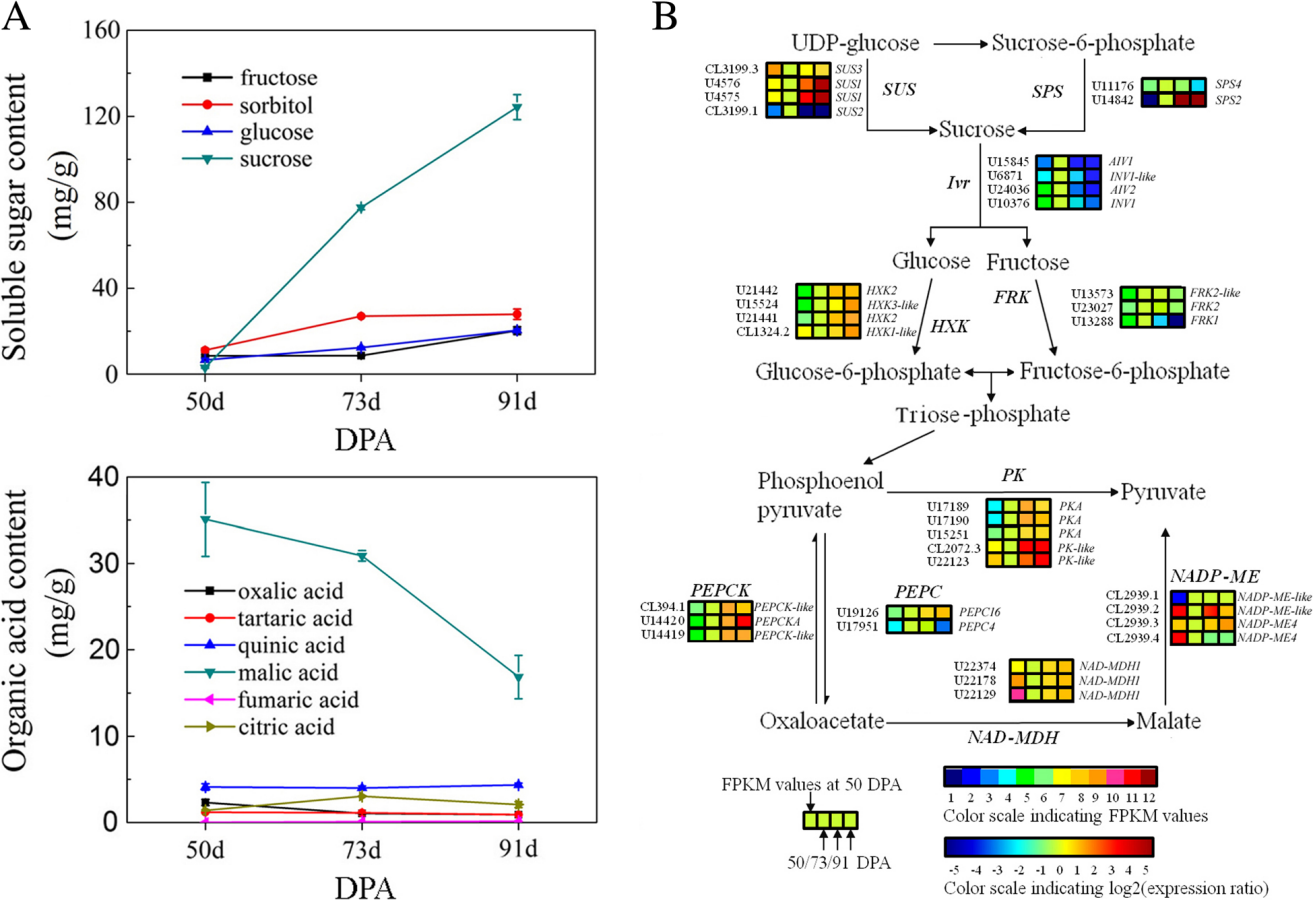
Changes in physiological and metabolic characteristics related to apricot fruit soluble sugars and organic acids during ripening. **a** Changes in content of soluble sugars and organic acids, **b** The biosynthetic pathway for sugars and organic acids. Enzyme names, unigene IDs and expression patterns are indicated. The expression pattern of each unigene is shown by 4 grids, with the left one representing the FPKM value at 50 DPA, and the second to fourth one from the left to the right representing the relative log2 (expression ratio) at 50 DPA, 73 DPA, 91 DPA, respectively. The grids show the absolute expression at 50 DPA, with the FPKM values 0–1, 1–2, 2–4, 4–8, 8–16, 16–32, 32–64, 64–128, 128–256, 256–512, 512–1024 and > 1024 represented by colors 1 to 12, respectively

Sucrose is the principal component of soluble sugars in apricot, and here we measured values ranging from 3.04 mg/g to 124.35 mg/g during fruit ripening, which makes up more than 60% of the total sugar content (Fig. 5a). One sucrose phosphate synthase gene (*SPS2*: U14842) and two sucrose synthase genes (*SUS1a*: U4576; *SUS1b*: U4575) were up-regulated, while two neutral invertases (*Ivr1-like*: U6871; *Ivr1*: U10376) kept downregulated (Fig. 5b, Additional file 5A, Additional file 6). Of eight SWEET genes identified, only one (*Sweet10*, U18631) was significantly up-regulated during fruit ripening. In addition, one sugar transporter gene (*STP13*, CL2738.2), two *STP5* genes (*STP5.1*, U18243 and *STP5.2*, U18242), and one early-responsive to dehydration gene (*EDR6*, C3054.1) showed significant increases in expression during ripening.

### Genes related to organic acid metabolism

The content of malic acid, which accounts for approximately 80% of total organic acids in apricots, decreased from 35.10 mg/g to 8.4 mg/g during ripening (Fig. 5a). The transcript levels of three unigenes (U22374, U22178, U22129), encoding NAD-malate dehydrogenases (*NAD-MDH1.1–1.3*) increased during the first two stages but remained stable during ripening. However, one NADP-dependent malic enzyme gene (*NADP-ME*: CL2939.3) and one phosphoenolpyruvate carboxylase gene (*PEPCK*: U14420), as well as *PK-like1* (CL2072.3) and *PK-like2* (U22123), which are all involved in malate degradation, showed substantial increases in expression (Fig. 5b, Additional file 5B, Additional file 6). At the same time, most components of ATP metabolism also showed increased expression in interactive pathways (iPath) during ripening (Additional file 5C). In the present study, four aluminum-activated malate transporters (*ALMTs*: U26645, U10981, U14799, CL2526.2) and one mitochondrial citrate transporter (U6882) were identified as putative organic acid transporters. Among them, only *ALMT9* (CL2526.2) was significantly down-regulated during ripening.

### Genes related to the metabolism of aroma volatiles

**Fig. 6:**
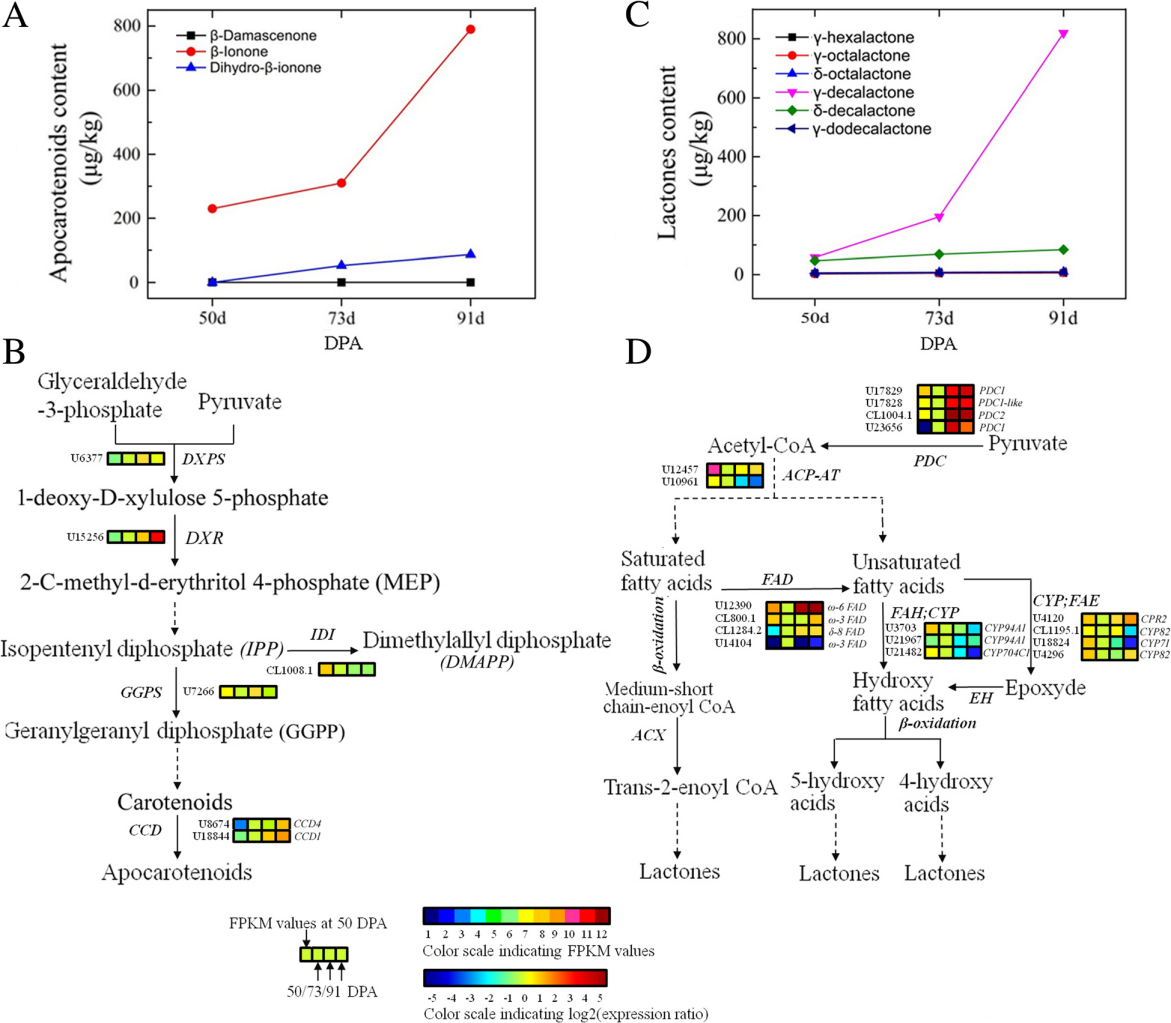
Changes in physiological and metabolic characteristics related to apricot fruit main aroma volatiles during ripening. **a**, **c** Changes in content of apocarotenoid aroma volatiles and lactones; (**b**), (**d**) The biosynthetic pathway of aroma apocarotenoids and lactones. Enzyme names, unigene IDs and expression patterns are indicated. The expression pattern of each unigene is shown by 4 grids, with the left one representing the FPKM value at 50 DPA, and the second to fourth one from the left to the right representing the relative log2 (expression ratio) at 50 DPA, 73 DPA, 91 DPA, respectively. The grids show the absolute expression at 50 DPA, with the FPKM values 0–1, 1–2, 2–4, 4–8, 8–16, 16–32, 32–64, 64–128, 128–256, 256–512, 512–1024 and > 1024 represented by colors 1 to 12, respectively

In the carotenoid cleavage pathway, β-ionone represented 90% of the three total apocarotenoid aroma volatiles identified (Fig. 6a). In the proposed biosynthetic pathways, the upstream 1-deoxy-D-xylulose-5-phosphate reductoisomerase gene (*DXR*: U15256) responsible for geranylgeranyl diphosphate (GGPP) synthesis was up-regulated, and the unigene U18844, encoding carotenoid cleavage dioxygenase1 (*CCD1*), responsible for apocarotenoid aroma volatile biosynthesis, was also significantly up-regulated during fruit ripening (Fig. 6b, Additional file 7). Lactone is the predominant aroma product in the fatty acid biosynthetic pathway, and we found that γ-decalactone, which increased rapidly during ripening, accounted for 85% of the total amount of lactone (Fig. 6c). In the proposed biosynthetic pathway, the expression of one of four differentially expressed unigenes, encoding fatty acid desaturase (*FAD2*: U12390), sharply increased during ripening. Similarly, up-regulated expression was observed for four fatty acid epoxidase genes (*FAE*: U4120) involved in lactone biosynthesis (Fig. 6d). In this study, among 13 ABC transporter G family members (ABCG) identified, unigene (*ABCG11*: CL2585.1) and unigene (*ABCG7*: CL929.1) were significantly up-regulated and down-regulated, respectively, during ripening.

### Genes related to hormone biosynthesis and signal transduction

The expression of some genes annotated as being involved in the biosynthesis and signal transduction of ethylene and abscisic acid (ABA) also changed during ripening. For example, we found that among 11 ACC synthase (ACS) and 15 ACC oxidase (ACO) genes identified in the study, *ACS2* (U12293) and *ACO1* (U22160) were significantly up-regulated. Seven ethylene-insensitive (ETR) unigenes were identified, but of these the transcript levels of only one (C186.3) decreased significantly. One ethylene-responsive element binding factors (ERF), *ERF010* (CL3301.1) was found to be up-regulated during ripening. Similarly, four AP2/ERF unigenes were found in apricot, with one (AP2D36) being down- and then up-regulated significantly during ripening.

One key ABA biosynthesis gene, 9-cis-epoxycarotenoid dioxygenase (*NCED1*:U19863), showed increased expression during ripening, while three phosphatase 2C protein genes (*PP2C11*: U4803; *PP2C27*: U19425; *PP2C33*: CL539.2) were down-regulated and an ABA-responsive transcription factor (*ABF2*: U6847) and a SNF1-related protein kinase 2 (*SnRK2*: U3561) were induced during ripening. In addition, a unigene (U24750) encoding an ABA receptor (ABAR) was highly expressed during ripening.

### Visualization of gene networks

**Fig. 7:**
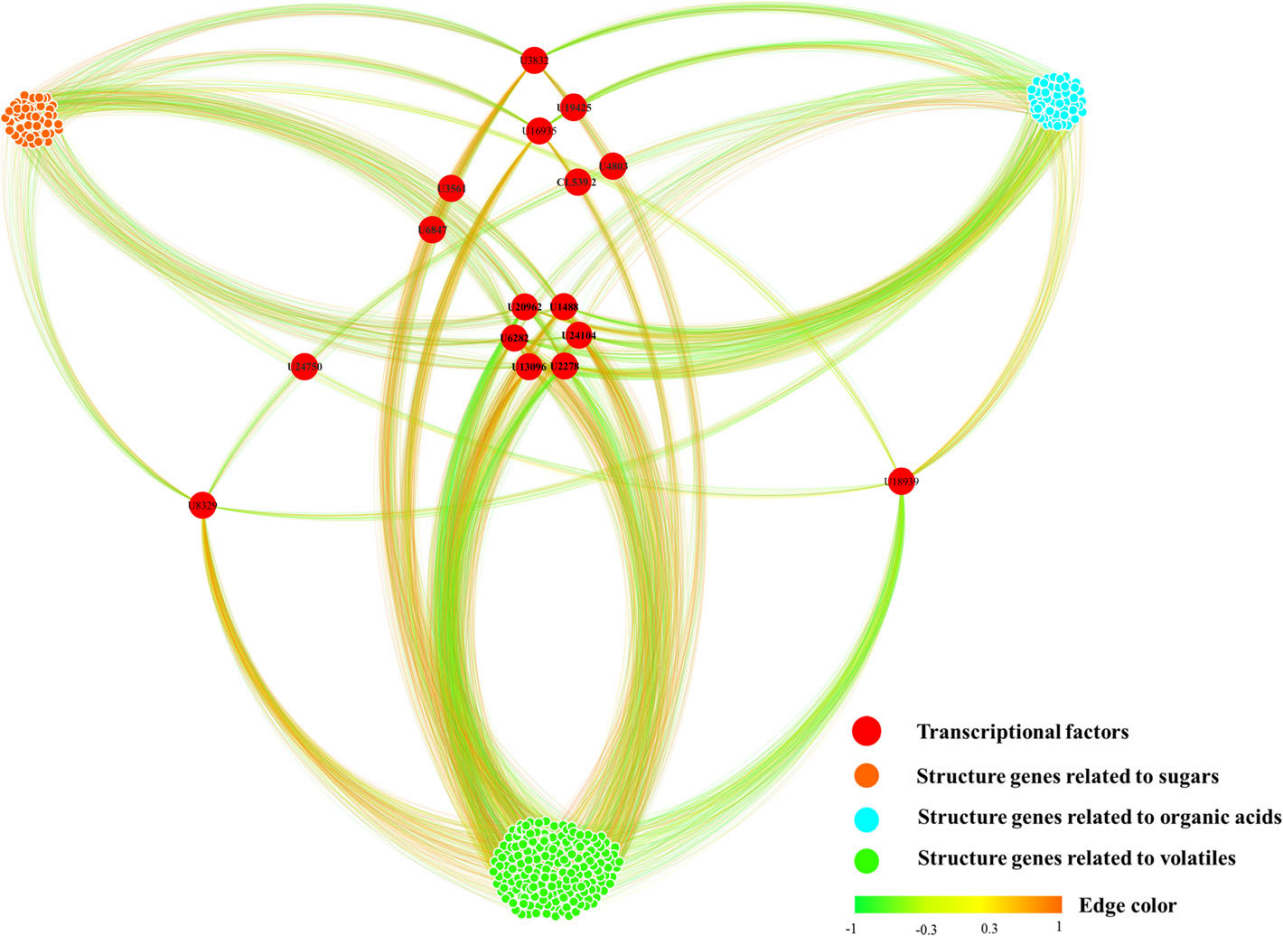
Co-expression network of transcription factors and structural genes potentially involved in the metabolism of sugars, organic acids and the main aroma volatiles. The network included 16 transcription factors and 143 structural genes from the “blue”, “cyan” and “red” modules presented in Fig. 4. Red dots indicate transcription factors, orange dots indicate sugar structural genes, cyan dots indicate organic acid structural genes, and green dots indicate volatile structural genes

To identify putative transcription factors related to flavor compound metabolism, the co-expression of candidate genes and regulators was visualized using the Cytoscape software platform (Fig. 7, Additional file 8). Among these transcription factors, four *ERFs* (*ERF4*: U16935; *ERF26*: U8329; *ERF12*: U3832*; AP2/ERF-like*: U18939) and six ABA signal elements were identified as hub genes for flavor compound metabolism. In addition to ethylene signal transduction elements, several other transcription factors were identified as candidate ripening regulators. Examples include U20962 and U13096, which are homologs of *MYB98* and *MYB1R1* from *Solanum tuberosum*, respectively; U6282, which showed highest similarity to a jasmonic acid insensitive transcription factor, *MYC4*; U24104, which was annotated as a homolog of the *Arabidopsis thaliana* transcription factor MYC3_ARATH; and unigene1488, which exhibited high similarity to the *A. thaliana* transcription factor *bHLH14*. Finally, U2278 was identified as an Agamous-like MADS-box protein similar to AGL12 (MADS12) from *Arabidopsis*.

### Quantitative PCR (qPCR) confirmation of gene expression

**Fig. 8:**
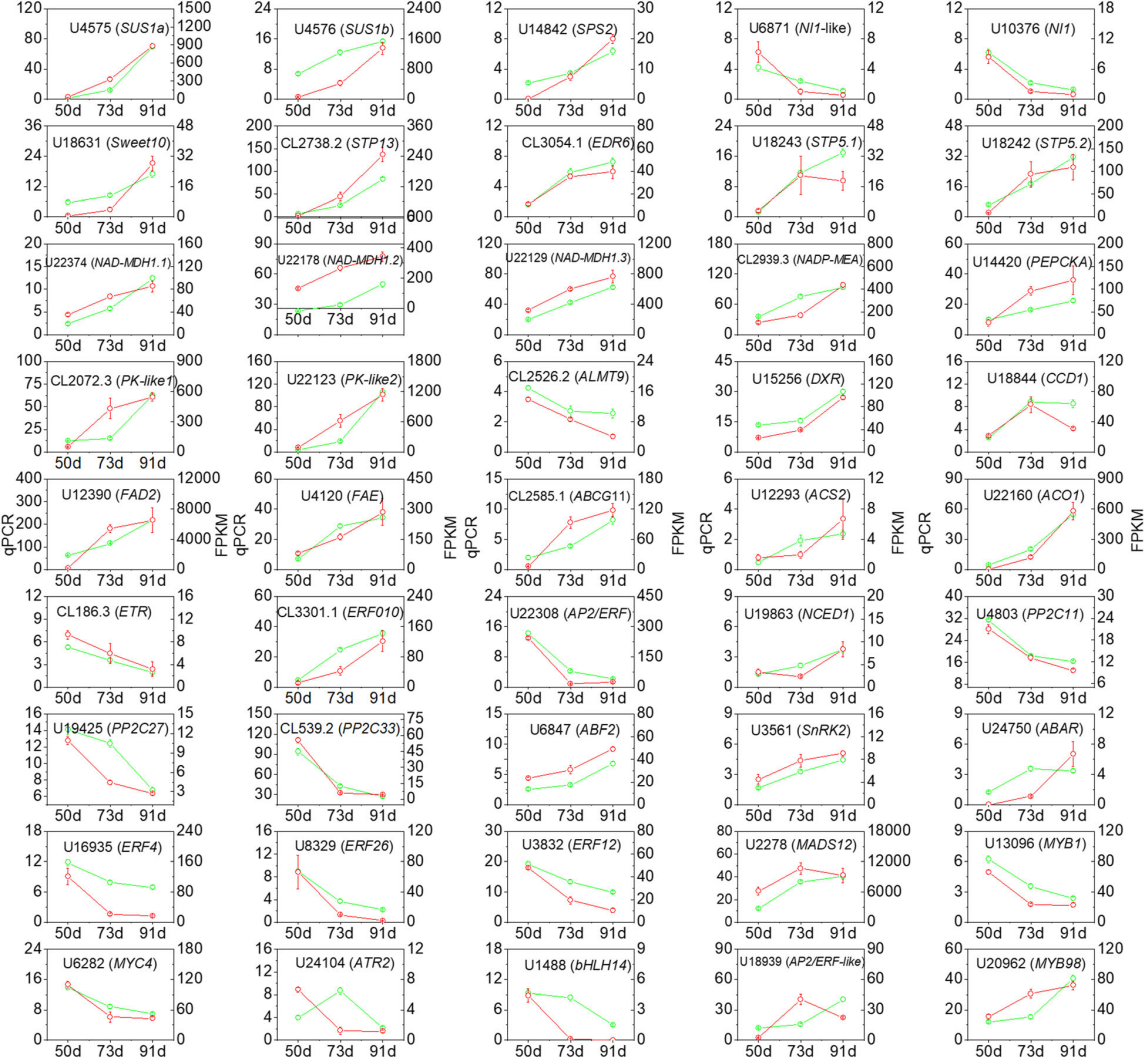
Confirmation of expression patterns for 45 genes involved in the metabolism of sugars, organic acids and main aroma volatiles using quantitative (q)PCR. The relative expression of target genes relative to a control gene (peach *ACT*) is shown with standard errors. qPCR values are shown as green lines, FPKM values from the RNA-seq experiment are shown as red lines

To validate the RNA-seq data, we examined the expression of a set of 45 candidate genes by qPCR (Fig. 8). These included ten genes involved in sugar metabolism, eight involved in organic acid metabolism, five involved in aroma volatile metabolism, seventeen ethylene and ABA biosynthetic genes and signal transduction elements, and sixteen regulators of flavor compound metabolism. The results from the qPCR analysis were consistent with the FPKM values obtained from the RNA-Seq data.

## Discussion

**Fig. 9:**
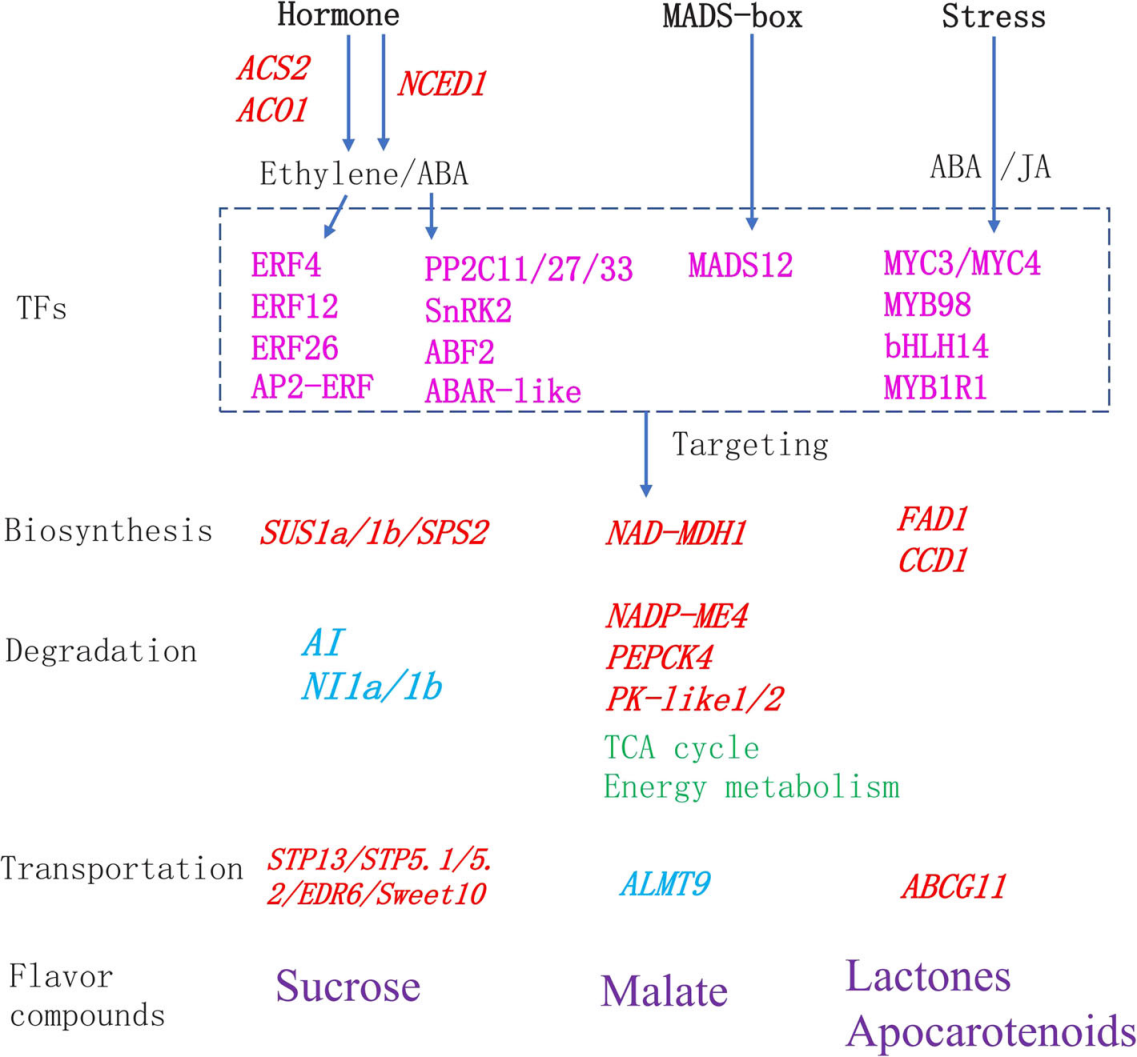
Schematic overview of the metabolism of the main flavor compounds during apricot fruit ripening. Up-regulated genes during ripening are indicated in red, down-regulated genes in blue; main flavor compounds are shown in purple, transcription factors in magenta. SUS, sucrose synthase; SPS, sucrose phosphate synthase; STP, sugar transporter protein; AI, vacuolar acid invertase; ALMT, aluminum-activated malate transporter; MDH, NAD-dependent malate dehydrogenase; NADP-ME, NADP-dependent malic enzyme; PEPCK, β-cyanoalanine synthase; PK, enolase; ABCG, ABC transporter G family member; FAD, fatty acid desaturase; CCD, carotenoid cleavage dioxygenase; ACS, ACC synthetase; ACO, ACC oxidase; NCED, 9-cis-epoxycarotenoid dioxygenase; PP2C, protein phosphatase 2C; SnPK2, SNF1-related protein kinase 2; ABAR, ABA receptor; ABF2*,* ABA-responsive transcription factors

Taste and aroma are important organoleptic properties of most fleshy fruits, as well as indicators of nutrition value for humans and animals. As with color, texture and nutritional quality, taste and aroma are generally shaped by coordination of developmental and biochemical pathways [13]. Previous studies have indicated that the main taste and aroma-contributing compounds in apricot change substantially in abundance during ripening [3], and that the transcriptome also undergoes major changes during this process [6, 21]. In most fruit, even no composition differences of sugar and organic acid are observed between flesh and peel, the contents of aroma volatiles are generally more abundant in peel than in flesh, peel attribute largely to the flavor of whole fruit, and thus it is the ideal tissue for investigating mechanisms of flavor quality formation in fruit [3, 4, 22]. In this study, transcriptome and metabolome-derived data were integrated to identify of genes related to flavor compound metabolism in apricot using fruit peels. We conclude that the production of flavor compounds in apricots is controlled by a combination of regulatory networks that control hormone signaling, developmental factors and stress responses (Fig. 9), providing a picture of metabolic pathways and regulatory networks related to flavor compounds.

Sucrose is the predominant sugar in apricot fruit, and its partitioning in fruits is mainly determined by sink strength [3], the accumulation is mainly related to the activity of sucrose-metabolizing enzymes, such as SPS, SS and neutral invertase (NINV) [23]. We found that *SPS2*, *SUS1a* and *SUS1b* genes were up-regulated, and propose that they are related to sucrose biosynthesis in the fruit. When sucrose is transported into the vacuole, it can also be converted to fructose and glucose by vacuolar acid invertases (VAINV). Accordingly, we observed that the *Ivrl1a* and *Ivrl1b* were up-regulated during ripening, which correlated with an increase in fructose and glucose levels. Generally, sugars and organic acids in fruit are stored in the vacuole [24], and the vacuolar accumulation of sugars requires many specific transporter proteins located in the tonoplast, such as sucrose transporters (SUTs/ SUCs), tonoplastic monosaccharide transporter (TMT), and sugar transporter protein (STP). In the present study, we observed that the expression levels of *Sweet10*, *EDR6, STP13*, *STP5.1* and *STP5.2* were upregulated and propose that these may contribute to the transport of sugars into the vacuole, or the adjacent recipient sink cells [25]. These results indicate that sugar metabolism shifts from synthesis to degradation during ripening in apricot, reflecting a balance between synthesis, degradation and transport.

HXK proteins are key regulators of glycolysis, and we found that two *HXK2* genes, two *HXK3-like* genes, and one *HXK1-like* gene were ripening up-regulated. Even though *NAD-MDH1*, which contributes to malate biosynthesis, was not down-regulated, *NADP-MEA*, *PEPCK4*, *PK-like1* and *PK-like2*, involved in malate degradation, showed substantial up-regulation. These results suggested that glycolysis was activated during fruit maturation. In the iPath, the TCA cycle, which can produce intermediates of organic acid metabolism, was also accelerated (Additional file 4C), indicating that the flux change from sucrose metabolism to organic acid metabolism was enhanced during ripening. Most of the genes annotated as participating in energy metabolism also showed increased expression in iPath (Additional file 4C), suggesting that more malate was consumed in the ATP production. Together with glycolysis, TCA cycle and energy metabolism, this could modulate the decrease in malate levels during fruit ripening. Like sugars, organic acids are mainly stored in the vacuole, a process that depends on specific transporters, such as vacuolar V-ATPase tonoplast dicarboxylate transporter pumps and members of the aluminum-activated malate transporter1 (ALMT1) family proteins [26]. In *A. thaliana*, *AtDT*, *AtALMT9* and *AtALMT6* were found to transport organic acids and recently, similar results were observed for four *AtALMT9* homologs in grape (*Vitis vinifera*) berries [27]. Two *ALMT-like* genes and an *ALMT II* gene were reported to be positively associated with fruit acidity in apple [28, 29]. Here, we identified a down-regulated malate transporter, *ALMT9*, that correlates with decreased acidity during apricot ripening.

Apocarotenoid aroma volatiles and lactones provide apricot fruit with the unique floral and fruity odors [3]. *CCD1* is correlated with apocarotenoid aroma volatile production in fruits [30]. In this study, a significant increase in apocarotenoid aroma volatiles, especially β-ionone, was detected during fruit ripening, and the *CCD1* gene was identified that may be involved in the biosynthesis of these compounds. *FAD* has been shown to play a central role in the desaturation of saturated fatty acids for lactone biosynthesis in peach (*Prunus persica*) [31] and strawberry (*Fragaria × ananassa*) [32], and here, we identified *FAD2* as a candidate gene for biosynthesis of these compounds. In recent, an ABC transporter was shown to facilitate the emission of volatile organic compounds in petunia flowers by transporting them from inside the cell to the outside [33]. We identified *ABCG11* was associated with this process in apricots.

The plant hormone ethylene plays a major role in the ripening and quality formation of climacteric fruit [34]. In this work, we observed that the expression of an *ACS2* and an *ACO1*, families of which are central ethylene biosynthesis, were significantly up-regulated during ripening, while the expression of *ETR*, an ethylene receptor, decreased significantly. ERFs are downstream ethylene signal transduction transcription factors, and AP2/ERF have been shown to be involved in the control of primary and secondary metabolism [35]. We found *ERF10* to be significantly up-regulated during fruit ripening, and one AP2/ERF [*AP2D36* (U22308)] to be down- and then up-regulated. Citrus *CitERF13* and *CitAP2.10* were shown to regulate citric acid accumulation and (+)-valencene synthesis, respectively [36], while *CitERF71* is involved in the synthesis of *E*-geraniol in sweet orange [37]. In addition to *ERF10* and *AP2D36*, the expression of specific *ERF* genes including *ERF4*, *ERF026* and *ERF12*, as well as an AP2-like *ERF* also correlate with flavor compound accumulation in apricot fruit. Recently, an ERF-MYB transcription complex was found to regulate furaneol biosynthesis in strawberry [38]. We identified an ERF interaction factor, *MYB98*, which may regulate flavor formation in apricot. ABA has been well documented to play a central role in the ripening of non-climacteric fruit, however, its regulation role underlying ripening of climacteric fruit is becoming clear [34]. Exogenous ABA has also been shown to accelerate the maturation of apricot [39]. Here, the expression of *NCED1*, which is involved in ABA biosynthesis, was highly up-regulated during ripening, suggesting that ABA biosynthesis and signal transduction was induced. In tomato, *SlPP2C1-RNAi* led to increased endogenous ABA accumulation and significantly accelerated fruit ripening and altered the expression of fruit ripening genes involved in ethylene release and cell wall catabolism [34]. In our study, *PP2C11*, *PP2C27*, *PP2C33*, *SnRK2* were down-regulated and *ABF2* was up-regulated during ripening, indicating that the PYR1-PP2C-SnRK2 pathway was activated, consistent with previous studies [21]. The ABA-responsive transcription factor MdAREB2 directly activates the expression of amylase and sugar transporter genes to promote soluble sugar accumulation in apple, suggesting a mechanism by which ABA regulates sugar accumulation [40]. An AREB-mediated ABA signal was reported to increase the concentration of hexoses and organic acids in tomato during fruit development [41], and ABF2, an ABRE-binding bZIP factor, is an essential component of the glucose signaling response [42]. Additionally, exogenous ABA can increase the accumulation of volatile compounds via ABRE proteins and the regulation of gene expression profiles in cherry [43]. These studies suggest that the *ABF2* and *ABAR* genes identified were associated with the regulation of taste and aroma compounds in apricot fruit.

Recently, analysis of the fruitENCODE data reveals three types of transcriptional feedback circuits controlling ethylene-dependent fruit ripening [44], as the hub of circuits, MADS-box transcription factors have been shown to be involved in regulating ripening and quality [45]. For example, *CsMADS6* directly regulates *LCYb1* and other carotenogenic genes to coordinately and positively modulate carotenoid metabolism. *LeMADS-RIN*, the first example of a MADS-box affecting ripening, has been extensively studied in tomato [46]. RIN directly targets lipoxygenase (TomloxC), hydroperoxide lyase (*HPL*) and alcohol dehydrogenase (*ADH2*), which are critical for the production of characteristic tomato aromas derived from the LOX pathway [47]. AGAMOUS-LIKE1 (TAGL1), the tomato ortholog of the duplicated *A. thaliana* SHATTERPROOF (SHP) MADS box genes, is necessary for fruit ripening. TAGL1 activity in ripening is executed through the direct activation of *ACS2* [46], and its over-expression results in higher lycopene accumulation, swollen sepals, and high accumulation of the yellow flavonoid naringenin chalcone [48]. We also identified an AGAMOUS-like MADS-box protein, AGL12, which is associated with regulation of flavor compound metabolism.

Stress can induce the accumulation of specific metabolites via hormone signal transduction pathways. In the study, a homolog of *MYB1R1* from *Solanum tuberosum* induced by drought was suggested to play a role in flavor formation via an ABA response. This is similar to a recent result which showed that a MYB protein regulates proanthocyanidin biosynthesis in strawberry primarily via regulation of ABA synthesis [16]. Besides ABA, jasmonate (MJ) has been found to be induced by stress and to mediate the production of volatiles. It is reported that linalool production was regulated by JAZs, which interacts with AP2/ERF, bHLH and WRKY to regulate plant secondary metabolism [49]. JA can induce the synthesis of sesquiterpenes via the signal transduction transcription factor *AtMYC2* binding to the promoters of AtTPS11 and AtTPS21 [50]. In the current study, we identified a jasmonic acid insensitive transcription factor *MYC4* and a homolog of *MYC3* from *A. thaliana* [51], implying a potential regulatory role in flavor compounds formation.

## Conclusions

In this study, we compared the transcriptomes of apricot fruit at three developmental stages to understand the development of taste and aroma during ripening. WGCNA results indicated that four modules were highly associated with flavor compound accumulation. Structural genes (369) were related to sugar, organic acid and aroma volatile metabolism. *SUS1*, *SPS2* and *Ivr* as regulation points in sugar biosynthesis, while *NADP-ME*, *PK*, the TCA cycle, and mitochondrial energy metabolism, were indicated as being central to the accumulation of organic acids. The expression data indicated that *FAD1* and *CCD1* are likely involved in the biosynthesis of lactones and apocarotenoid aroma volatiles, respectively. Five sugar transporters (*Sweet10*, *STP13*, *EDR6*, *STP5.1, STP5.2*), *ALMT9* and *ABCG11* presented significant association with sugar, malic acid and aroma volatile transport, respectively. In addition, sixteen hub genes (*ERF4*, AP2-like ERF, *ERF26*, *ERF12*, *PP2C11/27/33*, *SnRK2*, *ABF2*, *ABAR-like*, *MADS12*, *MYC3/MYC4*, *MYB98*, *bHLH14* and *MYB1R1*) were identified as potential regulators of the biosynthesis of flavor compounds. The flavor quality development of apricot fruit during ripening depends on the network comprising of both ethylene and ABA signaling, ripening factors and stress transduction. The set of genes identified here provides new insights into the putative pathways for flavor compound metabolism in apricot.

## Methods

### Plant material

‘Jianali’ fruit at turning (S1, 50 DPA), commercial maturation (S2, 73 DPA), and full ripe (S3, 91 DPA) stages were harvested from trees grown in an experimental orchard located in the National Fruit Tree Germplasm Repository, Academy of Xinjiang Agricultural Sciences, Luntai, Xinjiang, China (Fig. 1). After harvest, all fruit were immediately transported to the laboratory. Thirty average sized fruit with no mechanical damage were selected and ten were then used for TSS and firmness determination and the tissue from the others was divided into peels and flesh and frozen in liquid nitrogen. The tissue was stored at − 80 °C for further use, and three replicates were used for each sample.

### Transcriptome sequencing, assembly and annotation

Total peel RNA was extracted using the RNeasy plant mini kit (Qiagen, Hilden, Germany). Determination of RNA quality and quantity was performed using a NanoDrop spectrophotometer and denaturing agarose gel electrophoresis [52]. Only RNA samples with an OD260: OD280 > 1.80 and no discernible degradation were used for sequencing.

Triplicate samples of peels at each development stage were used to construct nine apricot transcriptome libraries (S1-A, S1-B, S1-C, S2-A, S2-B, S2-C, S3-A, S3-B, S3-C). Sequencing was conducted on the Illumina Hiseq 2000 platform at Beijing Genomics Institute (BGI), China, and paired-end (PE) sequencing technology was used, the read length is 100 nt.

Reads were cleaned and used for de novo assembly using Trinity (Release-20,130,225, https://sourceforge.net/projects/trinityrnaseq/) [53]. Since there was no releasing genome of apricot (*Prunus armeniaca* L.), the assembling was performed without the aid of a reference genome. Non-redundant unigenes were obtained using the sequence clustering software packages TGICL (Version: v2.1) and Phrap (Release 23.0). Unigenes with m > 70% similarity was given the prefix C and other singletons the prefix U. Unigenes were annotated using the NR, Nt, Swiss-Prot, KEGG, and COG.

### Transcriptome data analysis

The DEGs between two samples was identified using the algorithm developed by BGI staff according to the precious study [54]. The null hypothesis and alternative hypothesis to identify expressed genes between two samples are defined as following: H_0_ a gene have same expression level in two samples; H_1_ a gene have different expression level in two samples. X is denoted as number of fragments that can uniquely map to gene A. For each transcript representing a small fraction of the library, p(*x*) will closely follow the Poisson distribution. p($x\Big)=\frac{e^{-\uplambda}{\uplambda}^x}{x!}\left(\uplambda\ \mathrm{is}\ \mathrm{the}\ \mathrm{real}\ \mathrm{transcripts}\ \mathrm{of}\ \mathrm{the}\ \mathrm{gene}\right)$. The total fragments number of the sample 1 is N1, and total fragments number of sample 2 is N2; gene A holds x fragments in sample 1 and y fragments in sample 2. The probability of gene A expressed equally between two samples can be calculated with the following formulas: $2\sum \limits_{i=0}^yp\left(i|x\right)$(while $\sum \limits_{i=0}^yp\left(i|x\right)\le 0.5$) or $2\left(1-\sum \limits_{i=0}^yp\left(i|x\right)\right)$(while $\sum \limits_{i=0}^yp\left(i|x\right)\ge 0.5$). Here, $p\left(i|x\right)={\left(\frac{N_2}{N_1}\right)}^i\frac{\left(x+i\right)!}{x!i!{\left(1+\frac{N_2}{N_1}\right)}^{\left(x+i+1\right)}}$. Thousands of hypothesis tests were done, and the suitable *p*-value for individual test is not enough to guarantee low rate of false discovery, so multiple testing correction for each individual hypothesis testing was performed to guarantee the low false discovery rate (FDR) in whole. The DGEs between each of two samples (50DPA and 73DPA, 73DPA and 91DPA, 50DPA and 91DPA) were screened with both conditions that FDR threshold < 0.001 and an absolute log_2_Ratio value ≥1. The screening was performed by Excel (Version: 2010). Overall unigene expression patterns, excluding those with no significant expression changes as determined by FDR analysis, or with changes > 32 fold, were analyzed for determination of differentially expressed unigenes via value of log2(FPKM 73DPA/FPKM 50DPA) and log2(FPKM 91DPA/FPKM 73DPA). Furthermore, Gene Ontology (GO) classifications were compared between up-regulated and down-regulated unigenes using WEGO [55].

Interactive Pathways (ipath) analysis was carried out via the interactive pathways explorer (Version: 2.0, http://pathways2.embl.de/). Through KO (KEGG Orthology) id, the expression of a specific gene family was summed from all family members. To understand the dynamic changes and absolute expression magnitude during fruit ripening, twelve different colors were applied to indicate different unigenes FPKM values. To obtain accurate relative expression levels, another iPath figure was generated, where the standard of changes was FDR < 0.001 and the absolute value of log_2_Ratio was ≥1 as mentioned above. Metabolic pathways related to sugars, organic acids, ATP metabolism and terpenes were manually produced.

### WGCNA and gene network visualization

Co-expression networks were constructed using the WGCNA (v1.29) package in R [56]. Among the 16,168 genes, those with an averaged NRPKM from three replicates > 1 were used for the WGCNA unsigned co-expression network analysis. The modules were obtained using the automatic network construction function blockwise. Modules were made using default settings, except that the soft power was 16, min module size was 30, and the merge cut height was 0.25. The eigengene value was calculated for each module and used to test the association with flavor compound metabolism. The candidate genes network was visualized by cytoscape (2.8, USA).

### Real-time quantitative PCR

Total RNA (1 μg) from each sample was reverse transcribed using the PrimeScript 1st Strand cDNA synthesis kit (TaKaRa, Dalian, China). The relative expression of 45 candidate genes was determined by quantitative real-time PCR using a CFX 96 real-time PCR detection system (Bio-Rad, Hercules, CA, USA), using SYBR Premix Ex Taq (TaKaRa, Dalian, China). The primers for the reference gene and candidate genes are listed in Additional file 9. Peach *ACT* were used as an internal control to normalize small differences in template amounts according to Zhang et al. [12]. The PCR program was initiated with a preliminary step of 5 min at 95 °C, followed by 50 cycles at 95 °C for 10 s, 60 °C for 10 s, and 72 °C for 15 s. PCR products were melted at 95 °C for 5 s, and then at 65 °C for 1 min to determine the specificity of the primers. ΔΔCT was used to calculate the relative expression level of genes. Three different RNA isolations and cDNA syntheses from the same tissue for RNA-Seq were used as replicates to qPCR analysis.

### Determination of soluble sugars, organic acids and aroma volatiles

The content of soluble sugars, organic acids and aroma volatiles in the fruit peel was analyzed by high pressure liquid chromatography (HPLC) and gas chromatography–mass spectrometry (GC-MS) as previously described [3]. Soluble sugars were separated in a 5.0 M NH_2_ (4.6 mm × 250 mm) column (GL Sciences Inc., Torrance, CA, USA.) and detected with an RI-1530 refractive index detector (JASCO International Co. Ltd., Tokyo, Japan), organic acids were separated in an ODS C18 (4.6 mm × 250 mm) column (Beckman Coulter Inc., Brea, CA, USA), and detected with a 166 UV–VIS detector (Beckman Coulter Inc., Brea, CA, USA).

1.5 g of peel powder was homogenized with 3 mL saturated sodium chloride solution, and then 20 μL authentic *n*-hexanol and methyl myristate were added as the internal standards to quantify the volatile compounds. A solid-phase microextraction (SPME) needle with a 1-cm long fiber coated with a 65 μm layer of polydimethylsiloxane, and divinybenzene (Supelco Co., Bellefonte PA, USA) was used for volatile extraction. The identification and quantification of volatiles was performed on an Agilent 6890 N GC equipped with a FID detector and a DB-WAX column (0.32 mm, 30 m, 0.25 μm, J&W Scientific, Folsom CA, USA). The chromatograms and mass spectra were evaluated with GC-MS Postrun Analysis software (SHIMADZU, GC-MS-QP2010, Japan) The compounds were tentatively identified by comparing their mass spectra with those in the data system library (NIST08). The authentic standards of sugar and organic acid were dissolved in pure water, and aroma volatiles were dissolved in *n*-hexane, respectively. All flavor compounds were quantified according to standard curves of authentic compounds. Extracts from three replicate samples were analyzed for each developmental stage.

## Additional files

Additional file 1: Throughput and quality of apricot RNA-Seq data. (XLSX 10 kb)

Additional file 2: Principal component analysis (PCA) of transcriptome data. Three replicates per sample were analyzed. The percentages on the axes indicate the values explained by each PCA. (JPG 390 kb)

Additional file 3: Statistics of assembly quality. (XLSX 10 kb)

Additional file 4: Functional categorization of genes differentially expressed during fruit ripening based on the ‘biological process’ category in Gene Ontology. (JPG 9580 kb)

Additional file 5: Interactive pathway analysis during apricot fruit ripening. The green, red, blue, yellow, and pink lines indicate genes with non-significant expression change, up-regulated, down-regulated, up-down-regulated and down-up-regulated, respectively. The areas with light green background indicate the metabolic pathways related to sugars, organic acids and ATP. (A) Sugar biosynthesis, (B) Organic acid biosynthesis, (C) ATP metabolism. (PNG 482 kb)

Additional file 6: Expression annotation and functional annotation of unigenes shown in Fig. 5. (XLSX 20 kb)

Additional file 7: Expression annotation and functional annotation of unigenes shown in Fig. 6. (XLSX 18 kb)

Additional file 8: Expression annotation and functional annotation of unigenes shown in Fig. 7. (XLSX 38 kb)

Additional file 9: Primers used for real-time quantitative PCR validation of RNA-Seq results. (DOCX 17 kb)
