# Supplementary figures and images for: Transcriptional regulatory networks controlling taste and aroma quality of apricot (Prunus armeniaca L.) fruit during ripening
Source: BMC Genomics. 2019 Jan 15;20:45. doi: 10.1186/s12864-019-5424-8 (PMC6332858; doi:10.1186/s12864-019-5424-8)

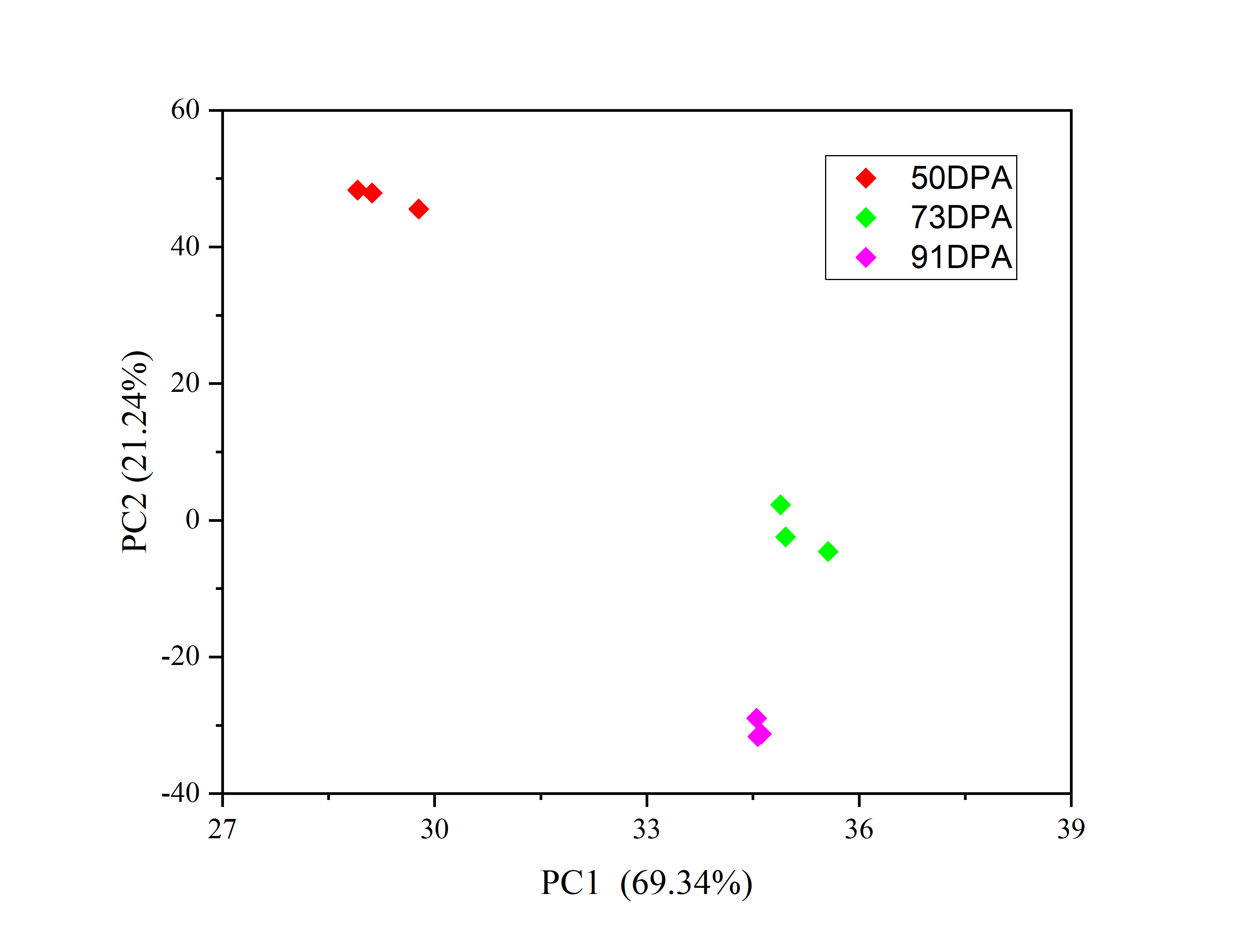

Supplement: Supplementary file 2 — Principal component analysis (PCA) of transcriptome data. Three replicates per sample were analyzed. The percentages on the axes indicate the values explained by each PCA. (JPG 390 kb) [file 12864_2019_5424_MOESM2_ESM.jpg]

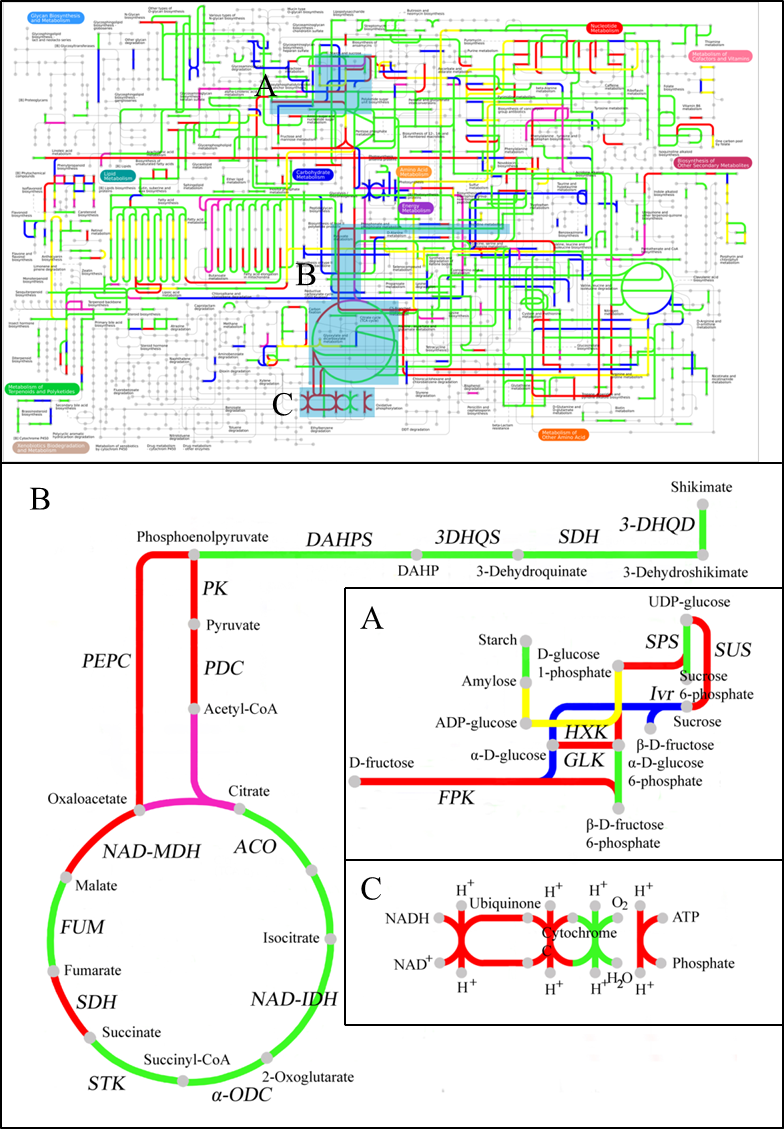

Supplement: Supplementary file 5 — Interactive pathway analysis during apricot fruit ripening. The green, red, blue, yellow, and pink lines indicate genes with non-significant expression change, up-regulated, down-regulated, up-down-regulated and down-up-regulated, respectively. The areas with light green background indicate the metabolic pathways related to sugars, organic acids and ATP. (A) Sugar biosynthesis, (B) Organic acid biosynthesis, (C) ATP metabolism. (PNG 482 kb) [file 12864_2019_5424_MOESM5_ESM.png]
