# Supplementary material for: Transcriptional regulatory networks controlling taste and aroma quality of apricot (Prunus armeniaca L.) fruit during ripening
Source: BMC Genomics. 2019 Jan 15;20:45. doi: 10.1186/s12864-019-5424-8 (PMC6332858; doi:10.1186/s12864-019-5424-8)
Supplement: Supplementary file 9 — Primers used for real-time quantitative PCR validation of RNA-Seq results. (DOCX 17 kb) [file 12864_2019_5424_MOESM9_ESM.docx]

Additional file 9. Primers for real-time quantitative PCR used for validation of RNA-Seq results.

| **Gene ID** | **Product Length (bp)** | **Sense Primer** | **Antisense Primer** |
| --- | --- | --- | --- |
| U4575 | 200 | CTCAACCGTCACCTTTCTGC | TGCTTGTAAGGGGTTTCTGG |
| U4576 | 292 | TTACTCTTCCTGGGCTTTACCG | TTGGCATTCTTACCATACCACTCA |
| U14842 | 170 | AAGGCTTGCTTGGTGGGT | CAGCGATGCTCTTATGTTTTCT |
| U6871 | 155 | TGATAAAGCCTTGGTGGA | ATCCCTTAGCAACATCTTCT |
| U10376 | 262 | TCCGCCACTATTCTCCCTG | CCACTCCCCTATGCTTCCTT |
| U18631 | 160 | TCCCTATTTCTTTTCTATGCCC | AGACAAGACAAATCCATCCCAC |
| C2738.2 | 199 | ATAGTGGAGGCAAGTCGTGTAGC | CGGCATAAAGGGAGGCATT |
| C3054.1 | 179 | ATGAGTTTCAGGGACGACAATG | TGGATAGGACCCAAAGCGAC |
| U18243 | 107 | CGAGGGCAAGATAACGGCTT | GCATTGTTGTCACTCCTCCTG |
| U18242 | 269 | CATCAAAATCCGAGGCAAGG | CCAACCCAAGAATAATAGACGC |
| U22374 | 122 | GAGGTTGTAGAGGCAAAGGC | GAGCACTCATAGACATCCCCAT |
| U22178 | 140 | AGGGTCTCACTGCCGCTAT | ACCAAACAACCTCTTTTCGTC |
| U22129 | 163 | ACATTGCCGTTATGGTTGG | TTGGTGTTTGCAGGATTAGC |
| C2939.3 | 197 | TGTTAATTGGAACGTCAGGAGC | TTCAACAGGCGGGAATGG |
| U14420 | 209 | AAGAAGGGCAATGGGATGT | GTCAGCGAAGCCAAAGATG |
| C2072.3 | 149 | GCCCGTTCTTTCCGTCGTT | CACTGGTAGACTCAGCAGGATGT |
| U22123 | 120 | TGATTGCTCTGTCTTTTGTTCG | TCGCCACCCCTTCCTGATT |
| C2526.2 | 273 | GTGTCACGATTCTTGCTTATTGC | AGCGTCCTCTTGGCTGGTA |
| U15256 | 238 | CCCGACAATGTGAAATACCC | CTCGTGCCCATAAATCGTAA |
| U18844 | 256 | AAAGTTTGCCCCAGTTGCT | GCTGTATTACCTGTCCCATTCC |
| U12390 | 201 | AAAGCCATCCCACCTCATT | CGCTATCACCCAAACACCA |
| U4120 | 229 | TTGACCACCTCCATTGCC | CGTATCGAGCTTTTCCTTCC |
| C2585.1 | 222 | CCAGAACGAAAACTATCCAAAG | CTCAATCTCCCTCCTAAAGCA |
| U12293 | 114 | AGTTCAGAAAGGCTGTGGCT | TGACCATCTCGTTCGCTCC |
| U22160 | 279 | CCTGTGATAGACTTTGGTGAGC | ATTAGAGGTGGGGCGATGC |
| C186.3 | 267 | CGCCTATTTCTCCATTCCC | TCCCGAGTTTTGACGCTTA |
| C3301.1 | 290 | TACAGAGGGATAAGGATGAGGAA | GGTGAGCGGTGAGCAGTTT |
| U22308 | 246 | AAGCCCAGGATGAATGTGAA | CCTTGGCTTCTTGCTAAACTCT |
| U19863 | 229 | CACCCCTTCCTCATCTTCAT | GGCGATTTGGACTTTTGG |
| U4803 | 162 | GAGGTGTTCTTGCCGTTTCC | AAGCCACAGCATCCTTATTTGA |
| U19425 | 259 | GTGCTCCCTTGAGTCTTCCC | AAGATGCCAATCACCTAATGC |
| C539.2 | 278 | GGTTCCTCTGATTTTGCTTCC | TGCTCGTTATCCTTGTGGTCT |
| U6847 | 104 | TTGGTCCCTCTTCTTGGTTAC | TGTTGGCTCTTGGGGTTGT |
| U3561 | 195 | TGGGAATGAAGATGAGGCA | CGAAAGCGATGAGTGAACC |
| U24750 | 208 | AAGAATGCCGAAGTGACAGAA | TTGGAGTGAGTTGGAAGGTTG |
| U16935 | 164 | CCAAGTCTCCAACCAAATCC | CTCCTCGGCAGTGTCAAAG |
| U8329 | 112 | GAGCCACGCAAGACCAAGA | TGAGAACAACATCGGCACCT |
| U3832 | 226 | GCCAAGGCCAAGACCAACT | CAACATTTCCCACAACACCAA |
| U2278 | 152 | AAGATCGAGAATCCGGTGCA | TTGGTGGCGAGTTCAAAGAG |
| U13096 | 139 | GCTGTTCTTGTTGGGTTTGC | TCGGTTGTGGTTGTTACGC |
| U6282 | 121 | GAAGATAATCGGATGGGACG | AGAAATGCTGGCATGGTGTA |
| U24104 | 251 | CATGGGGTGCAAGAGTGGA | TGGTTGGAATGTTGAAGAGGA |
| U1488 | 145 | CTATGCCCTCCGCTCTGTT | TTTCGGTTTCTGGGGTTGT |
| U18939 | 166 | CATAATGGAAGATGGGAGGC | TGATGTAACGGCTAAGGTCG |
| U20962 | 245 | AGACCAGCATAATGTCAGTAGCAC | TTCCATAACTGAGGATGTCGC |
